# *Ex vivo* comparison of V.A.C.® Granufoam Silver™ and V.A.C.® Granufoam™ loaded with a first-in-class bis-dialkylnorspermidine-terphenyl antibiofilm agent

**DOI:** 10.1016/j.bioflm.2023.100142

**Published:** 2023-07-11

**Authors:** Kaden B. Rawson, Travis Neuberger, Tyler B. Smith, Isaac J. Bell, Ryan E. Looper, Paul R. Sebahar, Travis J. Haussener, Hariprasada Reddy Kanna Reddy, Brad M. Isaacson, John Shero, Paul F. Pasquina, Dustin L. Williams

**Affiliations:** a Department of Orthopaedics, University of Utah, Salt Lake City, UT, USA; b Bone and Biofilm Research Lab, University of Utah, Salt Lake City, UT, USA; c Department of Biomedical Engineering, University of Utah, UT, USA; d Carle Illinois College of Medicine, University of Illinois, Urbana, IL, USA; e Department of Chemistry, University of Utah, Salt Lake City, UT, USA; f Curza Global, LLC, Salt Lake City, UT, USA; g The Center for Rehabilitation Sciences Research, Department of Physical Medicine and Rehabilitation, Uniformed Services University, Bethesda, MD, USA; h The Geneva Foundation, Tacoma, WA, USA; i Extremity Trauma and Amputation Center of Excellence, Joint Base San Antonio Fort Sam Houston, San Antonio, TX, USA; j Department of Rehabilitation, Walter Reed National Military Medical Center, Bethesda, MD, USA; k Department of Pathology, University of Utah, Salt Lake City, UT, USA

**Keywords:** Biofilm, Negative pressure wound therapy, MRSA, *A. baumannii*

## Abstract

Implementation of negative pressure wound therapy (NPWT) as a standard of care has proven efficacious in reducing both the healing time and likelihood of nosocomial infection among pressure ulcers and traumatic, combat-related injuries. However, current formulations may not target or dramatically reduce bacterial biofilm burden following therapy. The purpose of this study was to determine the antibiofilm efficacy of an open-cell polyurethane (PU) foam (V.A.C.® Granufoam™) loaded with a first-in-class compound (CZ-01179) as the active release agent integrated via lyophilized hydrogel scaffolding. An *ex vivo* porcine excision wound model was designed to perform antibiofilm efficacy testing in the presence of NPWT. PU foam samples loaded with a 10.0% w/w formulation of CZ-01179 and 0.5% hyaluronic acid were prepared and tested against current standards of care: V.A.C.® Granufoam Silver™ and V.A.C.® Granufoam™. We observed statistically significant reduction of methicillin-resistant *Staphylococcus aureus* (MRSA) and *Acinetobacter baumannii* biofilms with the CZ-01179 antibiofilm foam in comparison to current standard of care foams. These findings motivate further development of an antibiofilm PU foam loaded with CZ-01179.

## 1 Introduction

Negative pressure wound therapy (NPWT), also referred to as vacuum-assisted closure (V.A.C.), is a common pre- and post-operative therapeutic for orthopedic and soft tissue traumatic injuries sustained in the battlefield [1,2]. NPWT became popular among military physicians during Operation Iraqi Freedom (OIF) [1]: NPWT dressing use increased from 46% to 90% in traumatic wounds from March 2003 to September 2003 [3]. By 2007, NPWT was broadly applied in upper and lower extremity wounds in both OIF and Operation Enduring Freedom (OEF) by multiple branches of the armed services [3,4]. NPWT consists of a wound dressing, an adhesive barrier, a vacuum device with a collection canister, and specialized tubing to connect the wound site to the source of negative pressure. Polyurethane (PU) foam is one of the most commonly used dressing materials due to its biocompatibility and its large reticulations, which lead to thicker granulation tissue compared to gauze [5,6]. Therapeutic benefits are derived from increased perfusion to the wound site and concurrent removal of exudate due to the negative pressure applied by the vacuum [7,8]. Furthermore, vacuum-induced microdeformations promote increased tissue granulation and thus improve wound healing [9].

The nature of battlefield or other trauma-derived wounds make them highly susceptible to contamination, since they are often inundated with soil that hosts upwards of 5 x 10^9^ colony forming units (CFU) per gram [10]. Since 99.9% of bacteria in natural ecosystems, such as dirt and debris, are estimated to dwell in biofilm phenotypes, battlefield wounds are highly vulnerable to biofilm contamination at the point of injury [[11], [12], [13], [14]]. Furthermore, the majority of bacteria isolated from military wounds are capable of biofilm formation [15,16]. Characteristic components of bacterial biofilms include oxygen and other nutrient diffusion limitations for cells deep in the biofilm community [17,18]. This special subpopulation, also known as “persister cells,” display lowered metabolic activity, a trait associated with increased tolerance to common antibiotic compounds [19]. In some cases, the necessary dose to eradicate biofilms is thousands of times higher than clinical dosing and cannot be achieved due to toxicity limitations [20,21].

Methicillin-resistant *Staphylococcus aureus* (MRSA) and *Acinetobacter baumannii*—two of the ESKAPE pathogens—are known for their ubiquity in military and civilian wound infections. ESKAPE (*Enterococcus faecium, S. aureus, Klebsiella pneumoniae, A. baumannii, Pseudomonas aeruginosa,* and *Enterobacter species*) pathogens in general exhibit a growing multi-drug resistance and virulence problem, contributing heavily to hospital-acquired infection [22,23]. These infections pose an even greater risk in military settings where exposure to bacteria prior to wound care can be complicated by triage and transportation of soldiers [4,16,24]. *A. baumannii* was known for its malevolence in recent U.S. combat, earning its notorious reputation due to high prevalence during OIF and continued incidence in Veterans and Wounded Warriors who returned home [[25], [26], [27], [28]]. To mimic these challenging scenarios, a clinical strain of MRSA and *A. baumannii* ATCC BAA 1605 were used in this study.

In *Emergency War Surgery 5*th *Edition*, NPWT is indicated for a variety of therapeutic applications, including soft tissue and joint injuries, face and neck injuries, thoracic trauma, extremity fractures, and amputations as a means to “[maintain] an isolated wound environment” and “enhance the local wound environment and vascular permeability for wound healing.” Despite the touted benefits of vacuum assisted closure, military physicians are warned that “malfunction of NPWT devices can create an environment with a higher risk of infection.” To mitigate this potential risk, the textbook instructs that polymethylmethacrylate (PMMA) antibiotic beads laden with 1 g vancomycin and 1.2 g tobramycin per 40 g of PMMA may be applied beneath the NPWT wound dressing [29]. Beyond equipment failure, general risk of bacterial wound infection motivates improvements to antimicrobial strategies pertaining to NPWT for soldiers in transit to the next level of care.

Researchers and clinicians alike have long sought to improve NPWT-related healing by reducing bioburden in the wound site. Repeated instillation of known antimicrobials during NPWT shows promising reductions of bacteria in both *in vitro* and human trials [[30], [31], [32], [33]]. Physical and chemical alterations to PU foam are additional techniques that provide a degree of antimicrobial properties to NPWT wound dressings; among the most popular are silver- and zinc-based coatings [34,35]. In particular, silver has a long history and promising efficacy profile against planktonic bacteria [34,36,37]. However, there is a lack of evidence that supports the efficacy of these NPWT variations against ESKAPE pathogens in biofilm phenotypes. The significance of this clinical gap is highlighted by a study which isolated and cultured each of the ESKAPE microorganisms from biofilm within polyurethane and polyvinyl alcohol foams that were applied in both acute and chronic wounds [38]*.*

We showed previously that V.A.C.® Granufoam Silver™ has limited efficacy against established MRSA and *A. baumannii* biofilms *in vitro*, whereas V.A.C.® Granufoam™ loaded with CZ-01179 had significant antibiofilm potential [39]. However, efficacy profiles of each remained to be determined in an *ex vivo* setup.

We incorporated CZ-01179 into V.A.C.® Granufoam™ and compared its efficacy to V.A.C.® Granufoam Silver™ in a biofilm-contaminated *ex vivo* system. CZ-01179 was specifically identified through a collaboration with the University of Utah and Cūrza Global, LLC to expand upon a library of polyamine compounds, capable of eradicating biofilm-dwelling bacteria with broad-spectrum activity against Gram-positive and Gram-negative biofilms [[40], [41], [42], [43], [44]]. We hypothesized that V.A.C.® Granufoam™ loaded with 10.0% w/w CZ-01179 would eradicate biofilms of MRSA and *A. baumannii* by 3 log_10_ units more than V.A.C.® Granufoam Silver™ in an *ex vivo* porcine skin model in the presence of NPWT. The outcomes were anticipated to help determine whether CZ-01179-loaded foam could be translated toward subsequent *in vivo* testing.

## 2 Materials and methods

### 2.1 Materials

CZ-01179 was synthesized and provided by Cūrza Global, LLC. V.A.C.® Granufoam™ Large Dressing Kit (#M8275053/10), V.A.C.® Granufoam Silver™ Large Dressing Kit (#M8275099/10), ACTIV.A.C. ™ Therapy Unit, SENSAT.RA.C.™ Pad (#M8275057/10), V.A.C.® Drape (#M6275009/10), and ACTIV.A.C.™ 300 mL Canister with Gel (#M8275058/10) were purchased from 3M-KCI. Sodium hyaluronate (hyaluronic acid (HA)) was purchased from Lifecore Biomedical (#HA15M − 5).

FEP Tubing (1/16” ID x 1/8” OD, #EW-06406-62), Masterflex fitting (polypropylene, straight, hose barb union, 1/16” ID, #EW-06365-11), Masterflex L/S Digital Drive (#EW-07522-30), and Masterflex L/S (#EW-07534-08) were purchased from Cole-Parmer.

Heliplug collagen wound dressings (3/8” x 3/4”, #62–202) were purchased from Integra Miltex. Biofilm Reactor System (#CBR 90-1) with standard stir plate was bought from BioSurface Technologies Corporation. Clear 8-inch tempered glass dish (#KC35587) was purchased from Pyrex. Stomacher ® 80 microBiomaster Lab Blender (#0080/000/AJ/m) and Stomacher ® 80 Biomaster Strainer Bags (#030020580) were obtained from Seward. Tegaderm™ Transparent Film Dressing Frame Style, 15 cm x 20 cm (#70200749201) was purchased from 3M.

Scientific Freeze Dryer was obtained from HarvestRight, LLC. Brain heart infusion broth (#31FW55) was purchased from Grainger. Hardychrom™ Agar (#G307) was purchased from Hardy Diagnostics, and tryptic soy broth (#22092-500G) from Sigma-Aldrich. MRSA was a clinical isolate cultured from an infected knee [39]. *A. baumannii* ATCC BAA 1605 was obtained from the American Type Culture Collection.

### 2.2 Creating 10.0% w/w CZ-01179 antibiofilm foam

CZ-01179 was synthesized as part of a library of first-in-class antibiofilm antibiotics, inspired by naturally occurring peptides and aminosterols such as magainin and squalamine [40,45,46]. A 10.0% w/w concentration of CZ-01179 was selected based on previous experimental outcomes [31]. Antibiofilm foam samples were produced using previously published protocols [39]. In short, V.A.C.® Granufoam™ was utilized as the source of PU for the NPWT dressing. PU foam sections were cut into 4 cm x 4 cm x 1 cm rectangular prisms on a sterile field. HA was dissolved in PBS to produce a 0.5% (5 mg/mL) concentration and mixed at 500 rpm for 30 min. To facilitate a 10.0% w/w distribution of CZ-01179 within the foam scaffold, CZ-01179 was added to the 0.5% HA solution until a 6.64 mg/mL concentration was achieved. The hydrogel and antimicrobial combination were placed in a ∼2 °C refrigerator overnight. Samples loaded with 0.5% HA only served as comparative and process controls.

The 16 cm^3^ PU samples were submersed in the HA/PBS/CZ-01179 solution, compressed, and manipulated with a glass stir rod for 1 min to ensure complete saturation. Each sample was suspended with fine tip tweezers and rotated in air for 1 min to allow the excess hydrogel to flow out of the PU foam matrix. The saturated samples were secured to a Styrofoam block using five stainless steel rods, measuring 1.5 mm in diameter, placed through the center and each corner of a saturated PU sample such that it was suspended in air (no face of the foam touched a surface). Each foam sample was then frozen at −30 °C and lyophilized at 600 mTorr for 72 h. Samples that differed by more than +/- 5.0% from the target mass of 66.7 mg *active* CZ-01179 per PU piece, or a total weight of 666.9 mg, were not used for experimentation.

### 2.3 Biofilm growth and baseline quantification

MRSA and *A. baumannii* ATCC BAA1605 biofilms were grown for 48 h (24 h batch phase; 24 h flow phase) on collagen coupons (7 mg; 0.354 cm^3^) in a modified CDC biofilm reactor using previously established protocols [40,47]. Collagen coupons were used to mimic physiologically-relevant soft tissues [48]. Bioburden was quantified by removing a coupon from the CDC reactor with sterile forceps, placing it into 2 mL of sterile PBS, vortexing the sample for 1 min, sonicating at ∼42 kHz for 10 min, and plating on tryptic soy or Hardychrom™ (selective for MRSA) agar using a 10-fold dilution series following previously published procedures [47]. Agar plates were incubated for 24 h at 37 °C. Visible colonies were then counted and the CFU/coupon calculated.

### 2.4 Inoculation, incubation, and biofilm quantification of *exvivo* skin samples

Porcine skin was harvested from Yorkshire pigs at the time of a concurrent necropsy (pigs were from a separate study; IACUC approval was not required). Four cm x 4 cm excision skin samples were removed from the dorsal surface of the sacrificed pig along the epaxial muscle bed such that the fascial interface between the hypodermis and underlying tissue was preserved. Skin samples were cold sterilized for 30 min in 70% EtOH then 10 min in 10% bleach to eradicate normal flora on the epidermis and eliminate exogenous bacterial contaminants from tissue or the surrounding environment from the necropsy procedure.

Skin samples were rinsed with 1.5 L of 0.9% NaCl, then placed in sterile Petri dishes such that the epidermis contacted the bottom of the dish, and the preserved fascial interface was lying face-up. The rationale for this was, in a wound scenario, biofilms are likely to contact sub-epidermal and fascial layers as opposed to intact epidermis.

Biofilm-laden collagen plugs were removed from a modified CDC reactor with sterile forceps after the 48 h growth period and the biofilms were transferred to the basal dermal/fascial layer by rubbing a collagen coupon over a single quadrant (4 cm^2^) of the skin sample for 2 min. Following inoculation via rubbing, the spent collagen coupon remained on its respective quadrant. This process was repeated for each subsequent quadrant until the entire surface of the sample was covered/exposed to MRSA or *A. baumannii* biofilms.

Five mL of PBS was added to the well of the Petri dish to prevent the tissue from drying out and to maintain humidity within the growth environment. Samples were covered with the Petri dish lid and placed in an incubator for 48 h at 37 °C. This allowed biofilms to colonize the skin samples.

Following incubation, skin samples were gently rinsed with 500 mL of 0.9% NaCl to remove planktonic or loosely adherent cells. A 12 mm biopsy was collected from a randomly selected quadrant of each skin sample and placed in a Stomacher® 80 Biomaster Strainer Bag with 2 mL PBS and processed in a Stomacher® 80 microBiomaster Lab Blender at 120 rpm for 4 min. A 100 μl aliquot of the stomached solution was removed and quantified using a 10-fold dilution series as previously described. The bioburden was reported in CFU/g tissue and served as the “Post-48 h Incubation” baseline comparison for subsequent antibiofilm testing. Over the course of the study, a total of n=8 biopsies were quantified as “Post-48 h Incubation” baselines for each bacterial species.

### 2.5 Validation of biofilm growth on *exvivo* skin samples via scanning electron microscopy

Incubated skin samples were placed in individual, sterile petri dishes. 10.0% NBS formalin was pipetted into the dishes to the level of 1 mm beneath the horizontal surface of the sample, as to not wash away the biofilm. Samples were covered and placed in a sterile tissue culture hood for 24 h for fixation. Next, the formalin was evacuated from the petri dishes and the lids were left slightly ajar to air-dry for 24 h in the same sterile hood. After the samples were adequately dry, they were gold-coated for conductivity. The same process was used to prepare biofilm-laden collagen plugs. They were then imaged in a JSM 6610 SEM under secondary electron imaging (10.0 kV, 52 spot size, 10 mm working distance) at 500 x and 3,000 x magnifications.

### 2.6 Antibiofilm efficacy testing

**Fig. 1:**
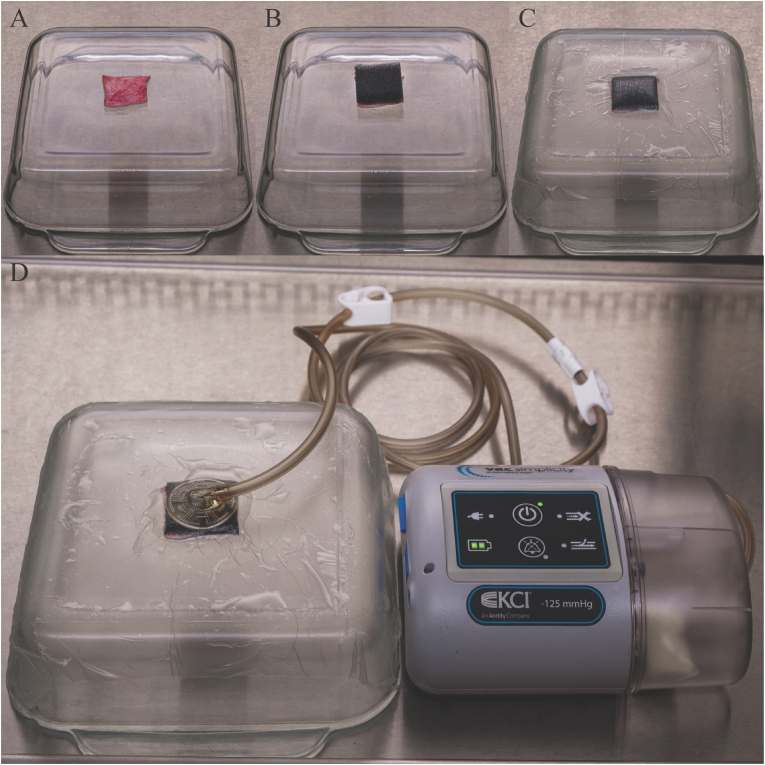
Photograph of NPWT test setup. A.) Inoculated porcine samples placed on sterile glassware; B.) NPWT foam cut to the size of the sample and placed overlapping inoculated sample; C.) Semi-occlusive adhesive placed over sample; D.) Complete model with NPWT device applied and engaged (−125 mm Hg).

Antibiofilm efficacy testing was performed in a tissue culture hood to maintain a clean environment. After the Post-48 h Incubation biopsy was aseptically collected and processed, the skin sample was transferred to an upside-down autoclaved 8-inch tempered glass baking dish (Fig. 1A); the steep, lateral faces of this testing surface allowed for adhesion of the semi-occlusive drape and simulated contours possibly found on live subjects. A 4 x 4 x 1 cm foam sample was placed on top of a 4 x 4 cm skin sample (Fig. 1B) and secured in place with a 15 cm x 20 cm adhesive Tegaderm™ film (Fig. 1C); the film ensured an airtight seal with the vacuum unit and tempered glass dish.

**Fig. 2:**
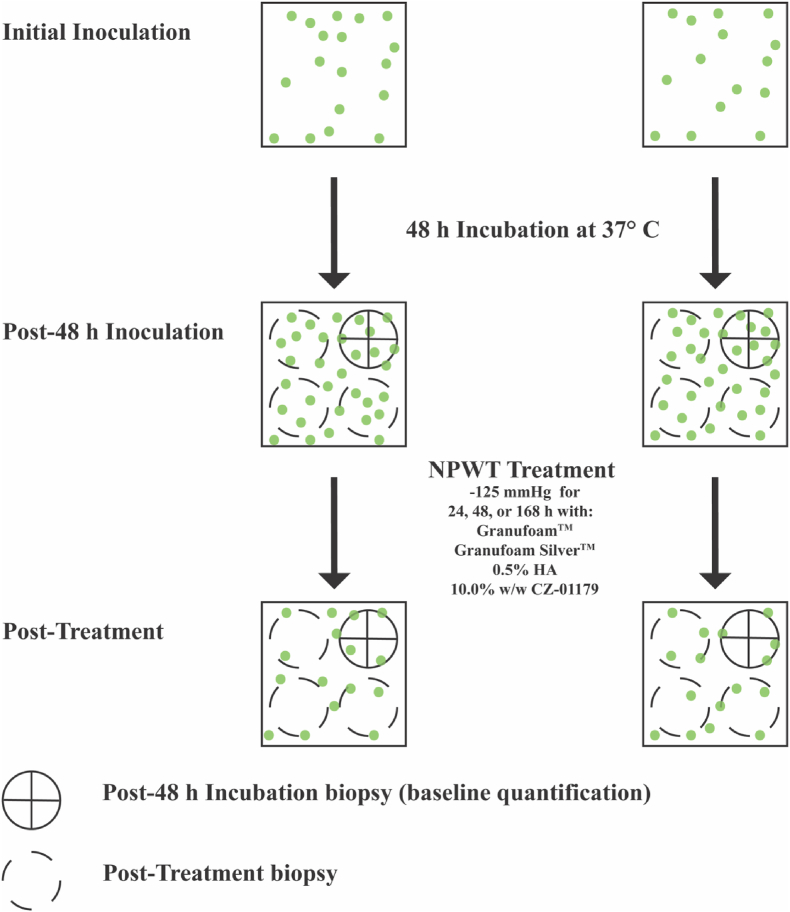
Flow diagram showing the process of inoculation, incubation, sampling, treatment, and quantification. Each 4 cm x 4 cm skin sample was divided into four equal quadrants. A 1 cm biopsy (circle with X) was taken from a random quadrant of each sample to determine the baseline bacterial load following inoculation and 48 h of incubation. After treatment with NPWT foam, the remaining three quadrants were biopsied and quantified to determine remaining CFU/g tissue.

The V.A.C® Drape was then placed across the testing surface, and a #10 scalpel was used to make two slits directly above the NPWT foam dressing. A SENSAT.R.A.C.™ Pad was adhered with the vacuum tube opening situated directly over the slits. The tubing was connected to an ACTIV.A.C.™ 300 mL Canister with Gel and ACTIV.A.C.™ Therapy Unit (Fig. 1D). Negative pressure was set for −125 mmHg and room temperature of ∼23 °C. Biofilm-ridden skin samples were exposed to each type of foam for 24, 72, or 168 h. The foam dressings, including adhesives and materials associated with the KCI-3M ACTIV.A.C ™ Therapy Unit, were exchanged every 24 h for those samples that were treated for 72 or 168 h. At the conclusion of each testing interval (24, 72, or 168 h), biofilm bioburden was quantified from the three remaining quadrants per sample using the aforementioned protocol such that a total of n=6 data points were collected per treatment (see Fig. 2).

### 2.7 Statistical analyses

Statistics were performed on GraphPad Prism 9 software. Averages and standard deviations were calculated. ANOVA was performed with Tukey's Multiple Comparisons test utilized when comparing treatments within the antibiofilm efficacy testing subsets, with alpha = 0.05.

## 3 Results

### 3.1 Outcome of loading 10.0% w/w CZ-01179 antibiofilm foam

**Fig. 3:**
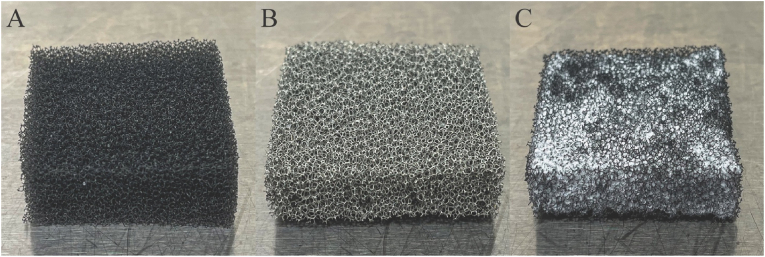
Images of PU foams with and without antimicrobials. (A) V.A.C.® Granufoam™ with no HA or CZ-01179. (B) V.A.C.® Granufoam Silver™. (C) V.A.C.® Granufoam™ loaded with 10.0% w/w CZ-01179 and 0.5% HA.

The process of loading HA and CZ-01179 into PU foam resulted in relatively uniform distribution of both materials across the foam sample (see Fig. 3). HA and CZ-01179 tended to accumulate mildly in the center of the foam but not to a degree that was considered preventative of proof-of-concept experimentation. Further, there was no indication that loading the PU foams with HA and CZ-01179 clogged or limited air or fluid flow as vacuum was drawn. Of more than 100 samples produced, approximately 5% were discarded as they did not meet the target mass of 66.7 mg active CZ-01179. The low discard rate suggested the loading method was reproducible.

### 3.2 Inoculation, incubation, and biofilm quantification of *exvivo* skin samples

**Fig. 4:**
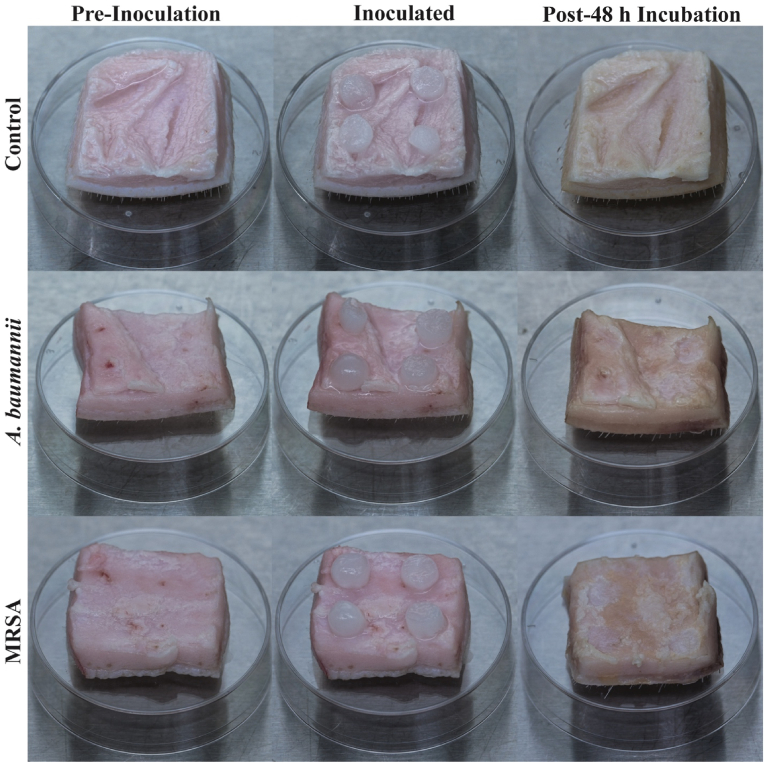
Appearance of porcine skin samples prior to inoculation (Pre-Inoculation), after initial inoculation (Inoculated) with four collagen coupons from the biofilm reactor, and after 48 h of incubation (Post-48 h Incubation). Samples in the control group were treated with sterile collagen plugs. The collagen coupons had nearly fully dissolved/eroded after 48 h of incubation.

Quantitative data suggested that MRSA and *A. baumannii* biofilms successfully transferred from collagen plugs and colonized the porcine skin samples. Collagen coupons that were used to obtain baseline CFU information had 9.30 +/- 0.42 log_10_ CFU MRSA/coupon. Skin samples inoculated with MRSA and incubated for 48 h had 8.57 +/- 0.42 log_10_ CFU/g of tissue. Meanwhile, collagen coupons with *A. baumannii* had an average of 8.34 +/- 0.37 log_10_ CFU/coupon. Skin samples inoculated with *A. baumannii* biofilms and incubated for 48 h resulted in a bioburden of 9.01 +/- 0.050 log_10_ CFU/g for the same time intervals on the sample tissue. Table 1 contains the specific inoculation and incubation bioburden for each treatment group, all of which were within one standard deviation of the reported averages in this section. Qualitative photographs portray the condition of the tissue samples before inoculation, after inoculation, and after 48 h incubation, as visualized in Fig. 4.

**Table 1:** Numerical representation of NPWT antibiofilm efficacy testing, expressed in log_10_ CFU/g, according to the study biofilm quantification protocol.

MRSA				*A. baumannii*		
		Log_10_ CFU/g	SD		Log_10_ CFU/g	SD
24 h	Inoculation	9.50 +/-	0.36	Inoculation	8.33 +/-	0.32
	Post-48 h Incubation	8.30 +/-	0.24	Post-48 h Incubation	9.03 +/-	0.57
	Granufoam™	8.31 +/-	0.47	Granufoam™	9.33 +/-	0.23
	Granufoam Silver™	8.94 +/-	0.42	Granufoam Silver™	9.17 +/-	0.23
	0.5% HA	8.83 +/-	0.63	0.5% HA	7.85 +/-	0.62
	10.0% w/w CZ-01179	6.98 +/-	0.18	10.0% w/w CZ-01179	5.12 +/-	0.47
72 h	Inoculation	9.10 +/-	0.32	Inoculation	8.37 +/-	0.36
	Post-48 h Incubation	8.56 +/-	0.31	Post-48 h Incubation	9.04 +/-	0.51
	Granufoam™	8.31 +/-	0.23	Granufoam™	9.03 +/-	0.09
	Granufoam Silver™	8.66 +/-	0.23	Granufoam Silver™	7.82 +/-	0.23
	0.5% HA	7.85 +/-	0.16	0.5% HA	8.66 +/-	0.81
	10.0% w/w CZ-01179	6.04 +/-	0.34	10.0% w/w CZ-01179	5.37 +/-	0.22
168 h	Inoculation	9.36 +/-	0.47	Inoculation	8.37 +/-	0.47
	Post-48 h Incubation	8.85 +/-	0.51	Post-48 h Incubation	8.95 +/-	0.50
	Granufoam™	8.62 +/-	0.19	Granufoam™	8.64 +/-	0.19
	Granufoam Silver™	9.22 +/-	0.28	Granufoam Silver™	7.61 +/-	0.26
	0.5% HA	7.51 +/-	0.46	0.5% HA	7.34 +/-	0.64
	10.0% w/w CZ-01179	5.12 +/-	0.20	10.0% w/w CZ-01179	0.00 +/-	0.00

### 3.3 Validation of biofilm growth ON SKIN samples via scanning electron microscopy

**Fig. 5:**
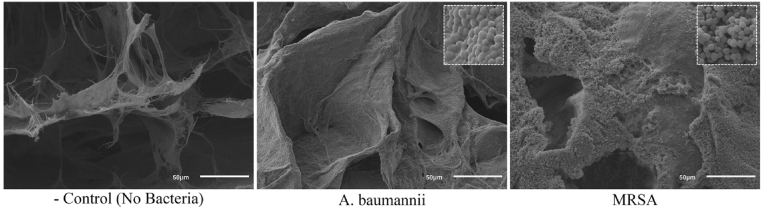
SEM images of MRSA and *A. baumannii* biofilm after 48 h growth in CDC reactor at 500 x magnification. Control included as reference for regular collagen structure.

**Fig. 6:**
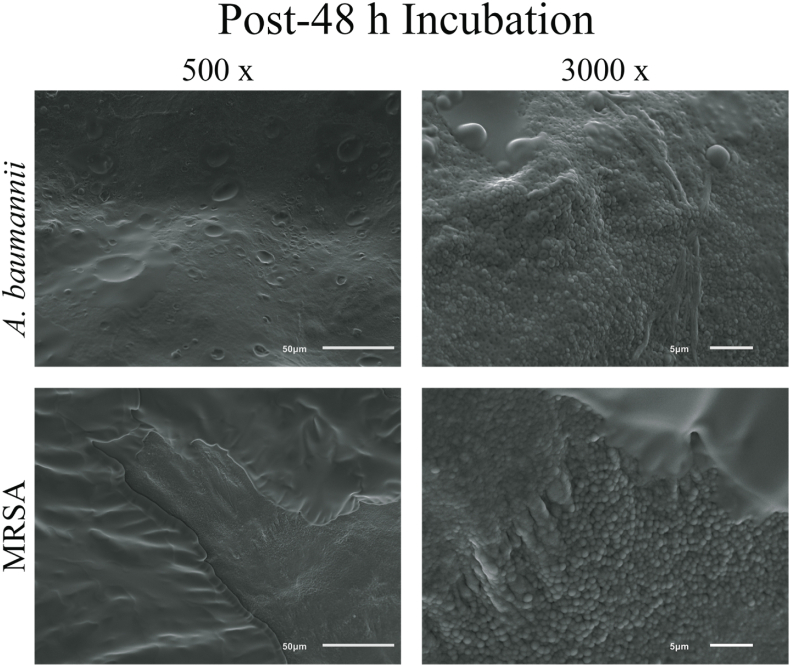
SEM images of porcine skin samples 48 h after inoculation with MRSA and *A. baumannii* biofilm at 500 x and 3,000 x magnification.

SEM imaging confirmed the presence of MRSA and *A. baumannii* biofilms on collagen coupons (see Fig. 5) and on *ex vivo* skin samples immediately following the 48 h incubation period (see Fig. 6). 500 x magnification portrays collections of MRSA and *A. baumannii* biofilms interspersed between melted collagen residues. 3000 x magnification provides a clear view of their three-dimensional, sheet-like structures of mature biofilm on the surface of the skin sample.

### 3.4 Antibiofilm efficacy testing

**Fig. 7:**
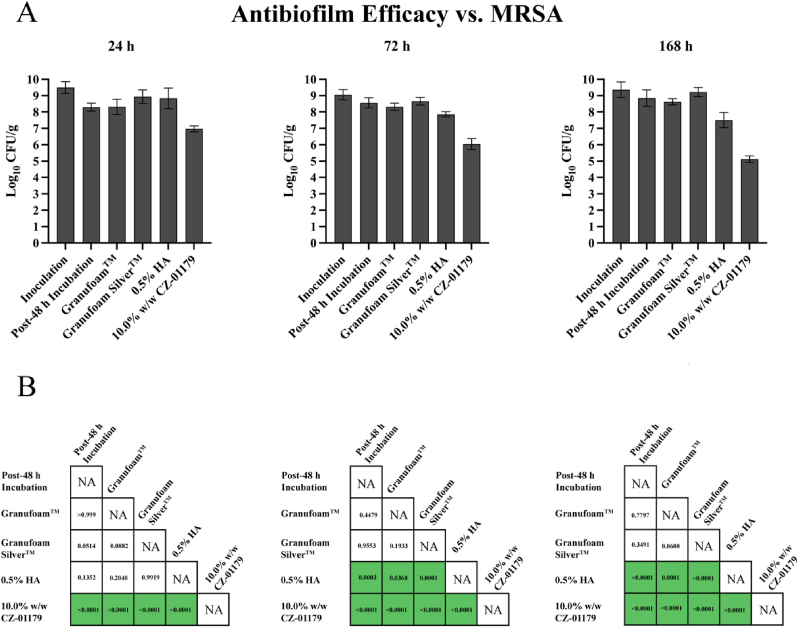
Quantification results for MRSA. (A) Graphical representation of NPWT antibiofilm efficacy in reducing MRSA biofilm, expressed in log_10_ CFU/g. (B) Results of Ordinary one-way ANOVA portraying statistical significance of multiple comparisons across all test groups according to respective treatment timeframes. Statistically significant values are highlighted in green (p < 0.05). (For interpretation of the references to colour in this figure legend, the reader is referred to the Web version of this article.)

**Fig. 8:**
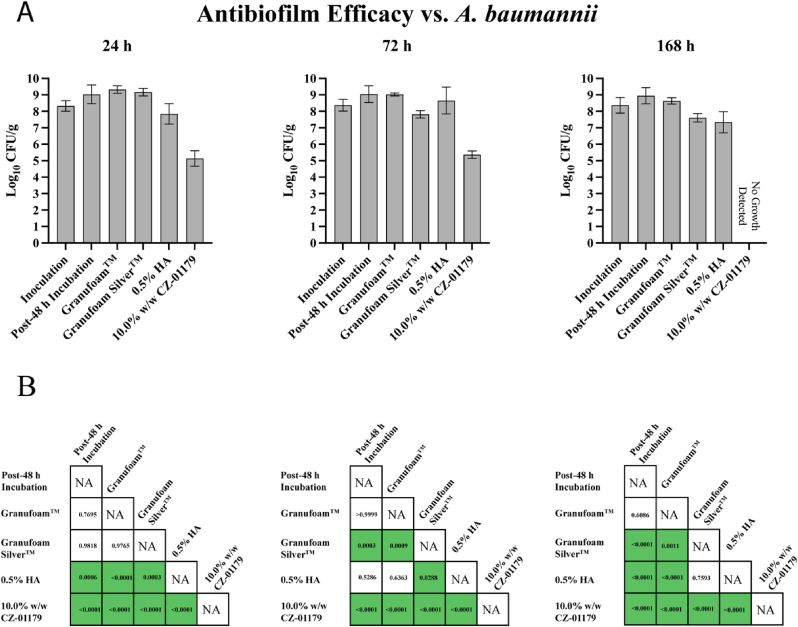
Quantification results for *A. baumannii*. (A) Graphical representation of NPWT antibiofilm efficacy in reducing *A. baumannii* biofilm, expressed in log_10_ CFU/g. (B) Results of Ordinary one-way ANOVA portraying statistical significance of multiple comparisons across all test groups according to respective treatment timeframes. Statistically significant values are highlighted in green (p < 0.05). (For interpretation of the references to colour in this figure legend, the reader is referred to the Web version of this article.)

Antibiofilm efficacy data outcomes for MRSA are graphically represented in Fig. 7 and *A. baumannii* in Fig. 8, while Table 1 shows the average CFU/g and SD at 24-, 72-, 168-h treatment intervals for each pathogen discussed in this section.

#### 3.4.1 MRSA

When compared to the Post-48 h Incubation, data showed that V.A.C.® Granufoam™ alone (PU with no antimicrobial agent) did not significantly reduce MRSA biofilms at any of the three time points. Specifically, the ΔCFU/g at 24 h was +0.02 log_10_ (p > 0.999), at 72 h it was −0.24 log_10_ (p = 0.45), and at 168 h it was −0.23 log_10_ (p = 0.78).

Skin samples treated with V.A.C.® Granufoam Silver™ had the opposite effect than what was anticipated; the silver-based foam primarily resulted in proliferation as opposed to reduction of MRSA biofilms when compared to Post-48 h Incubation controls. The ΔCFU/g at 24 h was +0.64 log_10_ (p = 0.051), at 72 h it was +0.10 log_10_ (p = 0.96), and at 168 h it was +0.37 log_10_ (p = 0.35). Interestingly, V.A.C.® Granufoam™ loaded with a lyophilized 0.5% HA scaffold without the addition of an antibiofilm agent showed greater antibiofilm efficacy against MRSA biofilms than V.A.C.® Granufoam Silver™. Specifically, the 0.5% HA-loaded foam resulted in a ΔCFU/g of +0.54 log_10_ at 24 h (p = 0.14), −0.71 log_10_ at 72 h (p = 0.0003), and −1.34 log_10_ at 168 h (p < 0.0001) compared to the Post-48 h Incubation baseline controls.

Notably, 10.0% w/w CZ-01179 resulted in a significant and notable reduction of MRSA biofilms across all treatments. Data showed that compared to the V.A.C.® Granufoam™ alone, foams loaded with CZ-01179 resulted in a ΔCFU/g of −1.34 log_10_ after 24 h of exposure (p < 0.0001), −2.27 log_10_ after 72 h (p < 0.0001), and −3.50 log_10_ after 168 h (p < 0.0001). Compared to V.A.C.® Granufoam Silver™, the CZ-01179-loaded foams reduced MRSA biofilms by significantly greater amounts. CFU/g tissue were reduced by 1.96 log_10_ (p < 0.0001), 2.61 log_10_ (p < 0.0001), and 4.10 log_10_ (p < 0.0001) CFU/g at 24 h, 72 h, and 168 h, respectively.

#### 3.4.2 A. baumannii

V.A.C.® Granufoam™ with no antimicrobial or HA had minimal activity against *A. baumannii* biofilms: ΔCFU/g was +0.29 log_10_ at 24 h (p = 0.77), −0.02 log_10_ at 72 h (p > 0.999), and −0.31 log_10_ at 168 h (p = 0.61) in comparison to the respective Post-48 h Incubation controls. V.A.C.® Granufoam Silver™ expressed some degree of efficacy against *A. baumannii* biofilms at 72 h and 168 h, with a ΔCFU/g of −1.22 Log_10_ (p = 0.0003) and −1.34 Log_10_ (p < 0.0001) compared to baseline. The silver-based foam did not, however, produce a reduction in bioburden at 24 h (Δ CFU/g = + 0.14 log_10_, p = 0.98).

The 0.5% hydrogel scaffold yielded mixed results: ΔCFU/g of – 1.19 log_10_ at 24 h (p = 0.0006), - 0.39 log_10_ at 72 h (p = 0.53), and – 1.61 log_10_ at 168 h (p < 0.0001). Data showed that when compared to V.A.C.® Granufoam™, 10.0% w/w CZ-01179 produced a ΔCFU/g of −4.19 log_10_ (p < 0.0001), −3.66 log_10_ (p < 0.0001), and −8.64 log_10_ (p < 0.0001) at 24-, 72-, and 168-h, respectively. When compared to V.A.C.® Granufoam Silver™, the CZ-01179 foams resulted in a ΔCFU/g of −4.04 log_10_ (p < 0.0001) at 24 h, −2.46 log_10_ (p < 0.0001) at 72 h, and −7.61 log_10_ (p < 0.0001) at 168 h.

## 4 Discussion

The concept of incorporating antimicrobials into wound dressings to reduce bacteria in wounds and promote healing is not unfamiliar to researchers or clinicians seeking to improve clinical outcomes associated with NPWT. Although PU-based products, such as V.A.C.® Granufoam™, have served as the standard of care since the popular adoption of VAC in wound care and management, materials such as polyvinyl alcohol (PVA) and silver-coated PU foams have also entered the marketspace. Recent studies suggest that PVA shows a significantly greater ability to reduce planktonic *P. aeruginosa* bacterial load than PU or silver-based foams [39]. Findings were in agreeance with those observed in this study: although silver-impregnated foams may mildly reduce bacterial burden, they fall short of clinical significance as defined by the 10^5^ rule [49,50]. Krizek found that 94% of skin grafts were successful when wounds were contaminated with less than or equal to 10^5^ CFU/g tissue [51]; Robson later showed that 28 of 30 wounds successfully closed when they contained 10^5^ or fewer bacteria per g [52].

Many attempts have been made to modify PU scaffolds; others have incorporated instillation protocols in conjunction with NPWT. The latter advocates for the use of antimicrobial solutions such as polyhexanide, acetic acid, or povidone-iodine in tandem with, or in alternating order to, the application of sub-atmospheric pressure [33,53]. One study with a solution of betaine/polyhexanide displayed successful wound closure before 6 weeks in 65% of complex infected orthopedic wounds [54]. Thus, agents with the ability to affect biofilm may have the greatest potential to advance NPWT strategies.

We also consider the inclusion of established biofilms in wound models to be imperative in the advancement of medical technologies that aim to manage biofilms. Ultimately, wound model creation can be divided into two stages: the method of biofilm growth and the chosen wound surface. We grew MRSA and *A. baumannii* biofilms in CDC reactors on a biocompatible surface according to established protocols that produce extracellular matrices similar to those observed in wild-type phenotypes to ensure the validity of our *ex vivo* antibiofilm analyses [41,47]. Significantly, this study demonstrated the ability to transfer established biofilms from a collagenous growth substrate to porcine soft tissue samples. Using biocompatible and bioabsorbable collagen allows translatability from *in vitro* to *ex vivo* to *in vivo* analyses using the same substrate [40,47].

Since this study was performed in preparation for a complex *in vivo* porcine model, pig skin samples were obtained from a concurrent project to provide an applicable and clinically relevant surface. As published throughout literature, research shows that porcine models most closely resemble human skin compared to their mammalian counterparts, especially in regard to features of tissue morphology and wound healing [[55], [56], [57]]. Bioburden quantification proved the marriage of established biofilm growth protocols with porcine skin samples as a clinically relevant model for soft tissue infection (Table 1).

After a suitable biofilm growth and transfer protocol was developed, application of NPWT provided additional insights into the antibiofilm efficacy of V.A.C. ® Granufoam™, V.A.C. ® Granufoam Silver™ and PU foam loaded with 10.0% w/w CZ-01179. Building on the results observed in our previous *in vitro* testing [39], V.A.C. ® Granufoam™ did little to reduce MRSA and *A. baumannii* bioburden at 24-, 72-, and 196 h increments in the presence of negative pressure. V.A.C. ® Granufoam Silver ™ allowed for growth and proliferation of MRSA biofilm across each of the 24-, 72-, and 196-h time frames compared to the baseline. However, since their corresponding p values are not statistically significant, we can infer that V.A.C. ® Granufoam Silver ™ expressed no antibiofilm efficacy against MRSA in the presence of NPWT. Notably, consistent with observations we have made in various studies over the years, the silver-based product performed better against the Gram-negative *A. baumannii* than Gram-positive MRSA, but while efficacy was observed, we wonder if the level of that efficacy would be sufficient to provide a clinically significant outcome; a query that further motivates *in vivo* work.

CZ-01179 continues to show its potential against biofilms in NPWT applications. At its shortest increment, 10.0% w/w CZ-01179 reduced MRSA and *A. baumannii* bioburden by 1.32 log_10_ CFU/g (p<0.0001) and 3.90 log_10_ CFU/g (p<0.0001), respectively, compared to corresponding baseline quantifications. Notably, 24 h of treatment with 10.0% w/w CZ-01179 greatly outperformed 196 h of treatment with V.A.C. ® Granufoam Silver ™ against the infamous *A. baumannii.* When examining the 196-h outcome of the study, CZ-01179 reduced biofilms to a far greater degree than other treatments. *In vivo* data will need to be collected to determine whether the levels of reduction observed with CZ-01179 would be clinically beneficial for *in vivo* wound healing. Notably, 0.5% HA possessed a marked degree of antibiofilm efficacy; it outperformed the V.A.C. ® Granufoam™ and V.A.C. ® Granufoam Silver™ against MRSA at 72 and 196 h. It likewise reduced *A. baumannii* bioburden to a greater degree at 24 h compared to the current clinical standards of care and performed comparable to V.A.C. ® Granufoam Silver™ at 1 week. These findings are corroborated by several other studies [58,59], but we observed that the greatest reduction was achieved with synergy between HA and CZ-01179.

As we look toward future iterations of the 10.0% w/w CZ-01179 in a 0.5% HA scaffold, we recognize areas of potential improvement. Increasing the size and scale of the simulated wound environments from 4 cm x 4 cm to more combat-relevant surface areas (∼5% of the body), our production techniques will face adaptation to accommodate the greater loads size of the pre-lyophilized HA/CZ-01179 solution in advanced shapes and conformations. Further, we will need to address the lack of homogenous distribution of the HA scaffold within the PU matrix. Current pooling is likely due to the slow migration of HA/CZ-01179 during the freezing process; however, a rotisserie-style rotational apparatus within the freeze dryer is under development to mitigate this outcome. Lastly, margins for cost of materials at scale will become tighter and incidental loss of CZ-01179 will be less tolerated to produce a scalable manufacturing process.

Future work is planned to assess the validity of the CZ-01179-loaded antibiofilm foam in an *in vivo* porcine wound model, similar to the work of Eriksson et al. [60] In addition to assessing the reduction in biofilm bioburden, *in vivo* work will assess variables such as wound closure, fluid removal from the wound site, and the presence of post-treatment immunohistochemical markers associated with biofilm infection.

## 5 Conclusion

There are limited options of antimicrobial NPWT foams or products on the market that drastically impact clinical outcomes. We seek to address this clinical gap by developing an antibiofilm PU foam that targets the biofilm phenotype. The data, showing that a 10.0% w/w CZ-01179 foam loaded in a 0.5% HA scaffold was more effective at eradicating MRSA and *A. baumannii* biofilms than the clinical standards of care, support and motivate future work with an *in vivo* porcine excision wound model.

## Funding information

Center for Rehabilitation Sciences Research Award, Grant/Award Number: HU0001-15-2- 0003.

## CRediT authorship contribution statement

**Kaden B. Rawson:** Conceptualization, Methodology, Validation, Formal analysis, Investigation, Data curation, Writing – original draft, Writing – review & editing, Visualization. **Travis Neuberger:** Conceptualization, Methodology, Validation, Investigation, Data curation, Writing – original draft. **Tyler B. Smith:** Investigation. **Isaac J. Bell:** Investigation. **Ryan E. Looper:** Resources, Supervision. **Paul R. Sebahar:** Resources, Supervision. **Travis J. Haussener:** Resources. **Hariprasada Reddy Kanna Reddy:** Resources. **Brad M. Isaacson:** Funding acquisition. **John Shero:** Funding acquisition. **Paul F. Pasquina:** Funding acquisition. **Dustin L. Williams:** Conceptualization, Validation, Writing – original draft, Writing – review & editing, Supervision, Project administration.

## Declaration of competing interest

The authors declare the following financial interests/personal relationships which may be considered as potential competing interests: Dustin L Williams reports a relationship with Curza Global that includes: equity or stocks. Ryan E Looper reports a relationship with Curza Global that includes: equity or stocks and funding grants. Paul R. Sebahar reports a relationship with Curza Globa that includes: equity or stocks, funding grants, employment, consulting, or advisory. Travis J Haussener reports a relationship with Curza Global that includes: consulting, employment, or advisory. Hariprasada Reddy Kanna Reddy reports a relationship with Curza Global that includes: consulting, employment, or advisory.

## Data Availability

Data will be made available on request.
